# Post-infection immunocomplex glomerulonephritis and Legionnaires' disease in a patient with adult Still's disease during treatment with interleukin 1 receptor antagonist anakinra: a case report

**DOI:** 10.1186/1752-1947-5-299

**Published:** 2011-07-09

**Authors:** Dieter Scholtze, Zsuzsanna Varga, Alexander Imhof

**Affiliations:** 1 Department of Internal Medicine, University Hospital Zürich, Raemistrasse 100, CH-8091 Zürich, Switzerland; 2 Institute of Surgical Pathology, Department of Pathology, University Hospital Zürich, Schmelzbergstrase 12, CH-8091 Zürich, Switzerland

## Abstract

**Introduction:**

Legionellosis is a systemic disease that primarily affects the lungs. However, dysfunction in many organ systems, including the kidneys, has also been described. There are only a few reported cases of renal dysfunction in patients with legionellosis.

**Case presentation:**

A 27-year-old Caucasian woman with known adult Still's disease was admitted to our hospital for community-acquired pneumonia, due to *Legionella *infection, with acute renal failure. Although her respiratory symptoms responded well to antibiotic treatment, her renal function worsened, with severe proteinuria and edema. A renal biopsy showed extracapillary and endocapillary proliferative glomerulonephritis with accompanying chronic and acute interstitial nephritis. This was consistent with a post-infection immunocomplex glomerulonephritis. After initiation of steroid therapy, her renal function improved. Additionally, therapy with diuretics and an angiotensin-converting enzyme inhibitor was initiated because of persistent proteinuria. Under this treatment regimen, her severe edema and proteinuria disappeared.

**Conclusion:**

To the best of our knowledge, there is only a handful of reported cases of post-infection glomerulonephritis with a nephrotic syndrome in a patient with legionellosis. Our findings suggest that, in patients with Legionnaires' disease with renal failure, post-infection immunocomplex glomerulonephritis should be considered and steroid therapy may be an effective modality to treat the renal complication.

## Introduction

Legionellosis is a systemic disease that primarily affects the lungs but can also cause dysfunction in many organ systems, including the kidneys. However, there are only a few case reports describing renal dysfunction in this clinical setting [1].

Post-infection glomerulonephritis (PIGN) is commonly seen as a complication of infection with nephritogenic strains of group A streptococci. Although Legionnaires' disease was also reported to be one of the infectious causes associated with tubulointerstitial nephritis worldwide, only a handful cases of PIGN have been reported [2,3].

## Case presentation

A 27-year-old Caucasian woman was brought to the emergency room of our hospital because of newly developed tachycardia with palpitations and hemoptysis. Our patient was receiving regular treatment for her Still's disease in our hospital's Department of Rheumatology. One week before her presentation to our emergency room, she was hospitalized in the Department of Rheumatology because of arthralgia and cough. The symptoms of cough and arthralgia were interpreted to be in keeping with her Still's disease and were treated symptomatically. During the next week at home, her arthralgia disappeared, but she developed swelling of both legs. The swelling worsened dramatically during the last days before her hospitalization. Our patient has a personal history of penicillin allergy. At the age of 20, she had been diagnosed with adult-onset Still's disease. She underwent many different treatments for her rheumatological disease, including cyclosporine, methotrexate, etanercept, steroids, adalimumab, infliximab and leflunomide, but every therapy was limited by side effects and non-compliance of our patient. The best results were actually achieved with permanent steroid therapy. Two weeks before her hospital admission the rheumatologists started a new treatment with anakinra.

At the time of admission, our patient's daily medications were prednisolone 20 mg daily, indomethacin 75 mg daily, esomeprazole 20 mg twice daily, and calcium carbonate with vitamin D_3 _twice daily. In our emergency room, our patient was drowsy and anxious. Her weight was 63.4 kg. Her blood pressure was 170/110 mmHg, her pulse rate was 160 beats/min (regular), and her temperature was 38.8°C. Her breathing rate was 40 breaths/min. She had normal cardiac auscultation without any murmurs. Her jugular venous pressure was elevated, and she had severe edema. Crackles were heard over both sides of her lung. On palpitation her abdomen was soft and non-tender without any pathological findings. Her neurological examination showed no sensory or motor deficiency. An arterial blood gas analysis showed a pH of 7.45, partial pressure of carbon dioxide 3.53 kPa, partial pressure of oxygen 10.5 kPa, hydrogen bicarbonate 18.2 mmol/L, and oxygen saturation 97%. Her electrocardiogram revealed a sinus tachycardia.

Her laboratory results at the time of admission are shown in Table 1. Serology for hepatitis B and C virus as well as human immunodeficiency virus testing was negative. Immunological tests showed rheumatoid factors < 10 IE/mL (normal value, < 20 IE/mL), antinuclear antibodies ratio 1:160 (normal ratio, < 1:10), anti-native DNA 92 IE/mL (normal value, < 20 IE/mL), anti-smooth muscle antigen 3 E/mL (normal value, < 10 E/mL), anti-U1 small nuclear ribonucleoprotein 1 E/mL (normal value, < 7 E/mL), antineutrophilic cytoplasmic antibodies 1:160 (normal ratio, < 1:10), glomerular basal membrane antibodies < 10 E/mL (normal ratio, < 1:10 E/ml), neopterin 24.60 ng/mL (normal value, < 2.50 ng/mL), complement component 3 c 0.32 g/L (normal range, 0.90-1.80 g/L), complement component 4 0.07 g/L (normal range, 0.10-0.40 g/l), immunoglobulin free κ-chains 60.70 mg/L (normal range, 3.30-19.40 mg/L), and immunoglobulin free λ-chains 60.10 mg/L (normal range, 5.71-26.30 mg/L). The κ/λ quotient was within the normal range at 1.01 (normal range, 0.26-1.65). The sputum Gram stain showed no bacteria, but the polymerase chain reaction assay was positive for *Legionella pneumophila *in more than one sputum test. Our patient refused to undergo a bronchoscopy. Antigen testing of our patient's urine for *L. pneumophila *serogroup 1 was also positive.

**Table 1:** Laboratory results on admission

Laboratory tests	Values	Normal ranges
**Blood**		
Hemoglobin	8.1 g/dL	11.7-15.3 g/dl
Leukocytes	9.89 × 10^3^/μL	3.0-9.6 × 10^3^/μl
Neutrophils	8.67 × 10^3^/μL	× 10^3^/μl
Platelets	370,000/μL	143,000-400,000/μl
**Blood chemistry**		
Blood urea nitrogen	13.8 mmol/L	2.14-7.14 mmol/L
Creatinine	92 μmol/L	44-80 μmol/L
Sodium	146 mmol/L	136-145 mmol/L
Potassium	4.0 mmol/L	3.3-4.5 mmol/L
Chloride	110 mmol/L	86-110 mmol/L
Magnesium	0.60 mmol/L	0.65-1.05 mmol/L
Calcium	1.50 mmol/L	2.09-2.54 mmol/L
Phosphate	1.00 mmol/L	0.87-1.45 mmol/L
Albumin	26 g/L	34-48 g/L
Lactate dehydrogenase	606 U/L	240-420 U/L
Aspartate aminotransferase	39 U/L	10-35 U/L
Alanine aminotransferase	67 U/L	10-35 U/L
γ-glutamyl transpeptidase	165 U/L	5-36 U/L
Alkaline phosphatase	164 U/L	35-104 U/L
Pancreatic amylase	51 U/L	13-53 U/L
Lipase	27 U/L	13-60 U/L
Total bilirubin	8 μmol/L	< 17 μmol/L
C-reactive protein	46 mg/L	< 5 mg/L
Blood glucose	4.0 mmol/L	3.9-6.1 mmol/L
Thyroid-stimulating hormone	8.03 mU/L	0.16-4.25 mU/L
Total cholesterol	4.2 mmol/L	< 5.0 mmol/L
HDL* cholesterol	0.92 mmol/L	>1.0 mmol/L
LDL* cholesterol	2.2 mmol/L	< 3.0 mmol/L
Cholesterol:HDL cholesterol ratio	4.6	< 5.0
Triglycerides	2.35 mmol/L	< 1.7 mmol/L
Ferritin	235 μg/L	10-150 μg/L
Prothrombin time, INR*	1.0	< 1.2
Thrombin time	13/second	< 18/second
Activated partial thrombin time	26/second	26-36/second
Fibrinogen factor	4.9 g/L	1.5-4.0 g/L
Antithrombin III factor	115%	75-120%

*HDL: high-density lipoprotein; LDL: low-density lipoprotein; INR: international normalized ratio.

**Figure 1:**
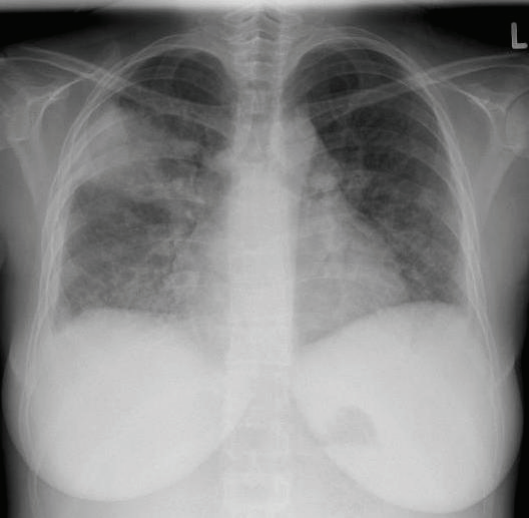
**Chest X-ray on admission day**.

In the first urine test, significant proteinuria and hematuria were conspicuous, but our patient was menstruating. Her chest X-ray revealed an infiltrate in her posterior right upper lung with small pleural effusion (Figure 1). Antibiotic treatment with levofloxacin (500 mg twice daily) was started.

Our patient's condition got better over the two days after therapy started, and her respiratory condition gradually improved. However over the next days, edema in both of her legs became worse and her weight (maximum weight, 68.4 kg) and creatinine level (maximum level, 121 μmmol/L) both increased. Her albumin level sharply decreased to 18 g/L. The repeated urine test still revealed severe proteinuria and hematuria. Her erythrocytes were dysmorphic, and there were some cylinders present (hyaline and granulated). Proteinuria of 6.76 g/24 h and albuminuria of 3112.4 μg/min were measured. A renal ultrasound showed bilateral renal enlargement with a reversed core-cortex border and normal vascular function.

**Figure 2:**
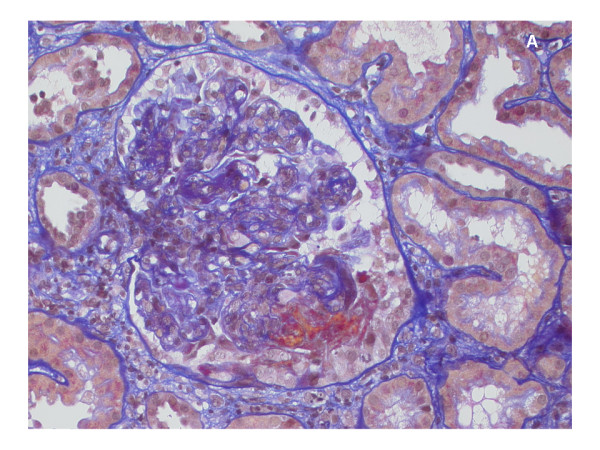
**Histological appearance of a glomerulum**. Hypercellular capillary loops and a larger extracapillary fibrocellular proliferation with evidence of necrosis (acid fuchsine orange G stain; original magnification × 400).

**Figure 3:**
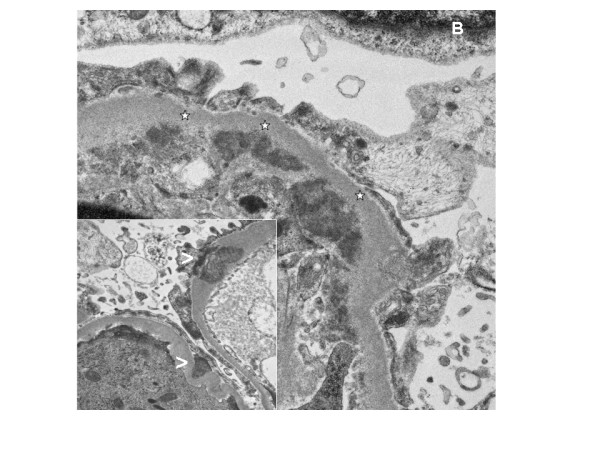
**Electron microscopy**. Ultrastructural appearance of a glomerular tuft. Several larger subendothelial deposits (*) and some larger subepithelial, hump-like (<) electron-dense deposits (original magnification × 400).

An ultrasound-guided renal biopsy was performed on her sixth day in hospital. The biopsy showed extracapillary proliferation in almost all glomeruli, with evidence of fibrinoid necrosis. The glomeruli displayed diffuse mesangial and endocapillary hypercellularity, and in several capillary loops there were neutrophilic granulocytes (Figure 2). Some mesangial areas showed mesangiolysis. No abnormalities of the glomerular basement membrane were seen using light microscopy. The accompanying interstitial changes revealed fibrosis and some degree of edema with lymphocytic infiltration. Tubules and vessels were without relevant pathological findings, especially without signs of vasculitis. An immunofluorescence examination showed mainly mesangial positivity for immunoglobulin G, complement component 3, complement component 1 q and immunoglobulin A, as well as κ- and λ-light chains. Electron microscopy confirmed the presence of subendothelial and mesangial electron-dense deposits, and in some capillary loops there were subepithelial, hump-like deposits. Podocyte feet effacement was diffuse (Figure 3).

These findings were consistent with an endocapillary proliferative immunocomplex glomerulonephritis with post-infection glomerulonephritis. Although our patient had no symptoms of streptococcal infection, we performed tests for antistreptococcal antibodies, all of which were negative (antistreptolysin O < 200 IE/mL, antihyaluronidase < 300 U/mL). So, we found no signs of a streptococcal infection.

Steroid therapy was initiated (prednisone 0.5 mg/kg body weight). Additionally, therapy with diuretics and angiotensin-converting enzyme inhibitors was started. After starting this treatment, our patient's renal function normalized, and over the next weeks her edema and proteinuria disappeared.

We identified a hot water boiler in the patient's apartment as the source of her *L. pneumophila *infection. The same *L. pneumophila *serogroup 1 infection that we found in our patient's sputum was found in the water of the boiler.

## Discussion

Although severe renal dysfunction associated with Legionnaires' disease has occasionally been reported worldwide, most cases are associated with tubulointerstitial nephritis, and only a few cases of PIGN in association with Legionnaires' disease have been described in the literature [1-3]. The mechanism of renal failure associated with Legionnaires' disease is mostly multifactorial. Histological examination of renal biopsy usually shows tubulointerstitial nephritis and/or acute tubular necrosis [4]. Among possible factors, those associated with dehydration or shock, rhabdomyolysis, endotoxemia and direct microbial toxicity were considered in our patient. In one previous report, the existence of *Legionella *bacteria was found by electron microscopy [5]. Therefore, recent reports describing the mechanism of renal dysfunction seem to point to direct renal toxicity from the *Legionella *organism or a systemic manifestation of Legionnaires' disease [5].

*Legionella*-associated acute renal failure has been described in case reports and small case series. Many of the cases reported in the literature were not accompanied by a renal biopsy, while others were confounded by severe hypotension, treatment with antibiotics that could cause acute interstitial nephritis, and severe multiorgan failure. Our patient developed acute renal failure without evidence of volume depletion, as evidenced by her stable blood pressure and physical examination findings consistent with euvolemia. It is a matter of common knowledge that various infectious diseases can cause renal dysfunction, such as *Streptococcus *infection, tuberculosis, yersiniosis, leptospirosis, Chlamydia, and different viral infections. The pathophysiology seems almost the same in all of these infections, including Legionnaires' disease [4,6].

In our case, interstitial nephritis was accompanied by an endo- and extracapillary proliferative glomerulonephritis. The mechanism of acute renal failure in legionellosis is not well understood. In the lung, the organism is phagocytosed into respiratory epithelial cells, where it replicates and induces cellular injury [7]. It is possible that the same process occurs in renal epithelial cells, both at the tubular epithelial cells and at the glomeruli. One previous case report supports this hypothesis. In that case, the *Legionella *organism was found by electron microscopy in the renal parenchyma of a patient with Legionnaires' disease [8]. In our patient, we could not identify the *Legionella *organism in the histological and ultrastructural examination, but the patient history and clinical findings implied a direct bacterial involvement in the pathogenesis of the disease. The elevation of antinuclear antibodies, antineutrophilic and anticytoplasmic antibodies and anti-native DNA was not significant. Light chains, which are in the family of uremic toxins, are often elevated in patients with acute or chronic renal failure [9]. The control of light chains after normalization of the patient's renal function two months after her initial presentation showed light chains in the normal range. Treatment with an interleukin-1 receptor antagonist may affect a person's susceptibility to the disease. These findings suggest that in patients with Legionnaires' disease who have renal failure, post-infection immunocomplex glomerulonephritis should be considered, and steroid therapy may be an effective treatment for this renal complication. A direct effect of anakinra for immunocomplex glomerulonephritis could also be possible, but we found no other cases in the literature to support this hypothesis.

## Conclusion

In summary, this case shows that *L. pneumophila *infection can lead to acute renal failure from post-infection immunocomplex glomerulonephritis. The mechanism is not wholly understood but may be due to direct renal toxicity. Glomerulonephritis less commonly presents as acute tubular necrosis or acute interstitial nephritis. When acute renal failure develops in a patient with Legionnaires' disease, tubulointerstitial nephritis is one possible cause, but immunocomplex glomerulonephritis should also be considered in rendering the differential diagnosis. Steroid therapy may be an effective modality for treating the renal complication; treatment of the other complications is also important.

## Abbreviations

PIGN: post-infection glomerulonephritis.

## Consent

Written informed consent was obtained from the patient for publication of this case report and any accompanying images. A copy of the written consent is available for review by the Editor-in-Chief of this journal.

## Competing interests

The authors declare that they have no competing interests.

## Authors' contributions

SD and IA analyzed and interpreted the patient data regarding the infectious disease and renal function. VZ performed the histological examination of the kidney. All authors read and approved the final manuscript.
